# Bibliographical Notices

**Published:** 1842-01-01

**Authors:** 


### Periscope;

OR,

### CIRCUMSPECTIVE REVIEW.

"Ore trahit quodcunque pòtest, atque addit acervo."

### Notices of some New Works.

Physiology for the Public. By Dr. *G. T. Hayden.* In a series of Monthly Numbers, price one Shilling each. No. 1, for Nov. 1841. Orr and Co., London—Fannin, Dublin.

Mankind have a natural curiosity to know the structure and functions of the human frame, and the attempt to teach such knowledge to the public at large, is much more rational and useful than the nature and treatment of diseases. Anatomy is an exact science, and may be readily learnt by any one. Physiology is fast approaching the same degree of certainty, and the broad outline of its laws may be safely and easily communicated to the world at large. Not so with pathology and therapeutics. These are little more than conjectural sciences —if indeed they deserve the name of science at all. These portions of the "ars medica" consist, as Baglivi long ago said, "entirely in observation of single cases." None but the experienced eye of the medical practitioner can detect diseases, and discriminate one from another. None but the experienced physician can safely select and apply the proper remedies—where there are remedies for the malady.

Hence it is, that a smattering of pathology and therapeutics invariably leads the non-professional man into errors, too often of a fatal character!! But a slight knowledge of the structure and functions of the living machine can do no harm in any case, and may not seldom enable the popular student of this class of knowledge, to ward off diseases by prudent precautions, and attention to the laws of Hygiene and Physiology.

Anatomy and physiology ought, indeed, to become constituent parts of a liberal education, whether that education be acquired in learned colleges or popular seminaries, and Dr. Hayden's plan of delivering lectures on physiology will probably be successful. A publication of these lectures will give them a wide circulation, as they are at a moderate price, and as much divested of technical phrases as the subject will allow. The first number, now before us, does little more than draw up the curtain, and therefore we are not in a position to form any very decided opinion as to the execution of the work. As far, however, as we can judge by the opening number, we are inclined to augur well of its successors. To the able lecturer and author we wish every encouragement; and hope he will be well rewarded for the pains he has taken to enlighten the public mind in a species of knowledge in which every individual is personally—we might say vitally concerned. We can make room only for one short extract as a specimen of the style of the author.

"Life, like its great Author, fills all regions—earth, air, and water. 'Professor Ehrenberg has discovered animalcules so minute that he computes each cubic line, which is nearly the bulk of a single drop, contains 500,000,000 of those monads, a number which almost equals all the human race.'

Now, when we conceive that life exists in each of these animals as certainly as in the largest, we are at once struck with the hopelessness of the search after that ethereal essence which, in one extreme, inhabits a microscopic animalcule, and in the other pervades the vast fabric of the huge whale or ponderous elephant. Still, it will be sought after, like the insane pursuit of perpetual motion.

. . . . . . . . . . . . 'The bubble on the stream,
That, in the act of seizing, shrinks to nought.' "

---

THE PROPOSED SCHEME OF MEDICAL REFORM IN REFERENCE TO CHEMISTS AND DRUGGISTS. By *G. Crook*, M.P.S. Price one Shilling. Hastings, London.

The plot thickens. Chaos is coming, if not come again! The mass of heterogeneous laws and regulations respecting the education, privileges, practice, &c. of the physicians, surgeons, and apothecaries of the British Isles, would seem inextricable, and almost irremediable. To add to the confusion—perhaps to cure it—a fourth class (the chemists) are fast raising their heads, and boldly prescribing a remedy which may be termed "*counter*-irritation." This prescription appears to be anything but palatable to the other grades of the profession—especially to the "GENERAL PRACTITIONER." The aid of Parliament, and of stringent laws is therefore to be sought forthwith, in hopes of curbing the *unlicensed* and unbridled application of this powerful agent. The more we have reflected on this subject, the more convinced we are that the evil is without any effectual remedy—at least in this country. Of all professions, avocations, trades, pursuits or callings, that of DOCTOR is the most universal. If you could *consult* every man, woman, and child between the Regent's Park and the Minories, you would not find *one* who could not prescribe some remedy for some disease—many of them for all diseases! Then again, every one prescribes occasionally—almost daily—for himself. A man eats a hearty supper of German sausages and sour beer. Next morning he finds himself tormented with the belly-ache, and repairs to the chemist's shop, where he consults the master or the man, as to whether he had better take a dose of castor oil—blue pill—or black draught. The chemist, after a moment's consideration, recommends him to take an ounce of castor oil, with peppermint water. No sooner said than done. The German sausages are ejected from the premises, and the man goes to his work—praising the "COUNTER-practice" and recommending it to his neighbour. Now this procedure, which is of daily and hourly occurrence, is as completely "medical practice," as if the patient went to the levee at Dr. Chambers' house, and, after waiting two hours and paying a guinea for advice, took the prescription to the same chemist, and paid eighteen pence or two shillings for a pill and draught, not perhaps, a whit better than the castor oil and mint water.

And are the chemists and druggists the only people who minister to the wants and wishes of the community in simple cases and in the remedial way? By no means. A man—perhaps a gentleman—eats an enormous dinner, and commits a debauch in the evening. Feeling head-ache and giddiness next morning, apoplexy rises in perspective, and he sends for Mapleson or some other operator, who seldom hesitates to abstract a pound of blood—without any rescript from Sir Henry Halford or Dr. Seymour. If this be not medical practice, and active practice too, we know not what "PRACTICE" means.

Another has had an apoplectic seizure followed by hemiplegia. The doctors and surgeons are slow in restoring the paralytic limbs, and the patient repairs to Argyle-street, where our friend LE BEAUME prescribes a six weeks' course of galvanism. Or he goes down to Brighton, where the Great Mahomet prescribes a dip in Medea's cauldron, from whence he is to come out in the vigour of youth.

In short, there is no end or limit to this extra-professional medication among all classes of society, from the peer to the pedlar. And then the Professed Charlatans! They are to be allowed not only to fill the newspapers with recommendations of their nostrums, but to sell them over the " counter," while the chemist is to be forbidden to recommend a dose of jalap or senna, or a blister, to those who come and ask his opinion as to the preference!

How is this universal medication to be prevented? Only by enacting a law to make it felony or transportation for any man to take a dose of physick, without prescription from a regular practitioner. That John Bull would strenuously resist this law as contrary to the spirit and safety of his "CONSTITUTION," no man, except the wildest theorist, can have the smallest doubt. The only practicable or probable remedy is an equalization of medical education for the three orders of the profession—an abandonment of pharmacy by the "GENERAL PRACTITIONER"—and a surrender of that branch to the chemist, whose education ought then to be regulated—when he will become, and indeed is fast becoming, the ci-devant, "APOTHECARY." The education of the "GENERAL PRACTITIONER" is now superior to that of the physician of former days and little inferior to that of the present M.D. Why then does he not relinquish the dispensing branch which must always be a connecting link with *trade*, and a source of great trouble, to himself, as well as of ungenerous suspicion on the part of his patients. This plan is practicable, because it exists in some of the most civilized nations of Europe, as, for instance, in France, Germany, &c.—nay it is acted on by a considerable number of general practitioners in England and Ireland, with infinite advantage. A law to charge for attendance, where medicine is only prescribed, not furnished, would we think, be obtained from the legislature—and indeed it only requires a general agreement among practitioners themselves to effect the change, without parliamentary enactments.

Unfortunately for physic, the perpetual wranglings, accusations, acerbity of language, recriminations, &c. which present themselves in almost every page of our periodical journals—in the newspapers—in courts of law—at coroner's inquests, &c. tend to lower the profession in the eyes of the public, and will greatly retard the passing of Acts for the amelioration of the members of that honorable calling! This is the bane of medical society—almost totally unknown in divinity or even in law. It is not very easy to account for this anomaly. No men, of any other profession, are so kind and liberal to each other, in the hour of sickness and distress—but that hour over, they are the thoroughest haters of one another on the face of the globe—a remark made by the late Cuvier! They seem never to dream that, in depreciating their neighbour, they are seriously injuring themselves in the long run. If there were some governing body, call it faculty, senate, council, or college, to regulate the education of the whole profession, and forming a court of appeal in cases of unprofessional conduct, much good would ensue—much obloquy and mal-practice would be prevented, and the general interests of the medical community guarded. As it is, we are a divided BODY, torn by dissentions, jealousies, and clashing opinions respecting medical polity. The prospect appears to darken, instead of clearing up.

---

BRANDY AND SALT—A REMEDY FOR VARIOUS DISEASES. By *J. H. Vallance.* Price 6d.

Morison's pills, that Lion or Leviathan of Allopathy—the lung-stretcher of Ely-place—mustard-seed—animal magnetism—nay homœopathy itself, may now hide their diminished heads. "BRANDY AND SALT" cure all diseases, and the remedy is within the reach of every individual, from a duke to a dustman. There never was a more ingenious invention, a more felicitous combination than "brandy and salt." The brandy makes the heart glad—and the salt increases the thirst for more brandy! Lucky invention—especially for the great promulgator, who has an extensive brandy-manufactory in France. None but the veritable *eau de vie* will have any virtue in combination with salt. Bett's and Booth's stuff are worse than useless—that produced in "LA FERTE IMBAULT," by W. Lee, Esq. is the elixir vitæ! Mr. Vallance appears to be the high priest of the temple of health in this country, and having been cured of a "bad leg," gratefully proclaims the following joyful tidings to suffering mortals.

"To all who acknowledge the superintending care of a DIVINE PROVIDENCE, the discovery of the remedy will appear as *a special gift from Heaven.*"

If this be not the height of blasphemy we know not what is! The way in which PROVIDENCE imparted this mighty secret to Mr. Lee, is somewhat remarkable. The estate of "La Ferte" was so much over-run with mosquitoes, and these little buzzing animals were so fond of English blood, that the proprietor had some thoughts of giving up his purchase, and returning to his native town of Leeds. But, just at this juncture, PROVIDENCE imparted to him the grand secret—the remedy, not only for mosquito bites, but for all the ills to which flesh is heir! Mr. Lee was too great a philanthropist—and too religious withall, to let this precious communication from the Deity remain unproclaimed to the nations of the earth. The "LEED'S MERCURY" had the honour of heralding the glad tidings, and now the pamphlet of Mr. Vallance will spread the "providential discovery"—we beg pardon—the divine annunciation, to the ends of the earth! Messrs. Lee and Vallance, however, are extremely candid and honest. They frankly admit that "brandy and salt" will probably fail in rendering man immortal, by reversing the decree—"dust thou art, &c." *See p.* 10. This is a "heavy blow and great discouragement" to the believers in the divine annunciation. There is one consolation, however, in the fact that, where the remedy fails to ward off death, it will, at all events, if freely employed, during life, preserve the body afterwards from corruption, and render it of little use to anatomists in their dissecting rooms.

The saturated solution of salt in brandy ("best French," mind) is to be taken at first, in the dose of a table-spoonful, early in the morning, mixed with water as hot as can be borne by the mouth and throat of the patient. In case of "worms or *paralytic* attacks," it is to be taken undiluted!! In order that all the glory of the remedy should be secured to the proper owner, it is strictly forbidden to take any other kind of liquor than the "brandy and salt" during the cure. This is a cunning law, for the remedy will be sure to be taken to the full extent of the prescription—"and something more"—as Lord Lyndhurst would say,—during the course of the treatment. We must admit, indeed, that there is some modesty in Mr. Vallance's pamphlet. For, while MORISON recommended his pills in *all* diseases, Mr. V. has only culled out forty of the principal afflictions to which mankind is liable, for the exhibition of "brandy and salt." Why he did not throw the remainder of the catalogue into the bargain is best known to himself—perhaps he did not know the names of the others, or found them rather too jaw-breaking, if he consulted Good's Nosology.

Among these "forty thieves" which daily rob us of the most valuable articles of our property—HEALTH, we find the following culprits who are sure to be exterminated by brandy and salt:—viz. gout, consumption! inflammation of the lungs, asthma, scrofula, palpitation, inflammation of the brain, cholera, insanity, cancer, "fevers of all kinds," paralysis, tic douloureux, spinal complaints, enteritis, mortification—and 24 other grievous maladies!! A large list of cases, cured of the various diseases enumerated in the pamphlet, is published at the end of the work. Mr. Vallance naively observes, in conclusion, that he has been at considerable expense in advertisements, &c. to make his remedy known—"*but very few of my correspondents recollected to enclose a remittance.*" He therefore pretty broadly hints that he will work no more *gratuitously* for an ungrateful public, and that those who chuse to apply to him for his pamphlet, must inclose six "PENNY STAMPS!" For actual advice, "ONE SHILLING" must be forwarded, which is to be carefully secured by wax or wafers, as the money has frequently oozed out, and came not to the hand of the philanthropic dispenser of a divine gift to mankind!

While Government permits, and while a community swallows such gross and unequivocal charlatannerie as this—and it is merely a sample—how preposterous is it to merge half, or rather three-fourths of our grievances in the "counter practice of the chemist and druggist!!"

---

On the Employment of the Microscope in Medical Studies. By *John Hughes Bennett,* M.D., Lecturer on Clinical Medicine, &c. Edinburgh, and London, pp. 27. Dec. 1841.

Pathology is divided, and usefully divided, into disorders of function and diseases of structure. The latter are, no doubt, the *consequences* of the former. As long as an organ shews no change in its material substance, when examined by the naked eye, we call its affections functional; but when visible or tangible alterations take place, we pronounce the complaint structural or organic—and too often beyond the remedial agency of our art.

It is highly probable, however, that the slightest *functional* disorder of certain organs and tissues, as the brain, the mucous membranes, &c. are attended, perhaps caused by, some minute changes in the organs themselves, not discoverable by the naked eye. If the microscope could be employed in such cases, it might throw some light on the subject. But how is it to be applied? Functional disorders are rarely fatal; and it can only be where death takes place from some other disease, that the functionally disturbed organ can be subjected to the microscope. The microscope, however, though it may not lead to so much useful knowledge, in respect to *therapeutics,* as the zealous students of histrology anticipate, reveals the minute structure of parts, whether healthy or morbid, in a manner which the naked eye could never attempt to emulate.

"To the naked eye, the brain and nerves appear to be composed of fibres. These have been demonstrated to you by your teachers. Whole volumes have been written in describing them, and numerous theories have been built upon the assumption of their presence. The microscope however tells us, as was first pointed out by Ehrenberg, that these supposed fibres do not exist, or rather that they all consist of numerous tubes, the walls of which are distinct, and contain a fluid which may be seen to flow from their broken extremities on pressure.

In looking at a muscle, the strongest sight can only detect fine longitudinal fibres, of which it appears to be made up. The microscope tells us that each of these fine fibres is composed of numerous smaller ones, and that these are crossed by other lines, which have received the name of transverse striæ. Formerly it was supposed that muscular contraction, the cause of motion in animals, was produced by the fibres being thrown into zig-zag rugæ. It has been shown from the microscopic researches of Bowman, that this appearance is owing rather to relaxation than contraction, which latter depends upon the approximation of the transverse striæ.

What disputes have taken place regarding the termination of the arteries, and the nature and supposed properties of capillary vessels! The microscope has shown us that a distinct net-work of vessels lies between the arteries and veins, partaking of the properties of either, and possessed of others peculiar to themselves. These have been denominated intermediary vessels by Berres, and serve to connect the arterial with the venous system. What on the other hand was understood by capillary vessels has been shown to have no existence.

On regarding with the naked eye the different glands, in which the secretions are formed, how complex they appear, how various in conformation, and how opposed to one another in structure. The microscopic researches of Malphigi, Weber, Müller, and others, have shown that they are all formed on one type; that the ultimate element of every gland is a simple sacculated membrane to which the blood-vessels have access, and that all glands are formed from the greater or less number, or different arrangement only of the primary structure.

The notion of most men respecting the skin is, that it is composed of epidermis, rete mucosum, and cutis vera. But it was by means of the microscope that Breschet, Vauzème, Gurlt, Simon, and others, discovered its real anatomy, and showed us the existence and relations of the papillæ, of the sudorific organs and their ducts, the inhalent and mucific apparatuses, and so on. All our knowledge of epidermic structures also, such as hair, horn, feather, &c., may be said to have originated in the use of the microscope.

In the same manner the real structure of cartilage, bone, tooth, tendon, cellular tissue, and, in a word, of all the solid textures, has been revealed to us,—so that it may be truly said, that all our real knowledge of structural anatomy, and all our acquaintance with the true composition of every organ in the body, has been arrived at by means of the microscope, and would never have been known without it."

Dr. Bennett, however, does not limit the utility of the microscope to the examination of minute structure in a healthy condition. Its application to morbid alterations is considered by him as still more important than to normal conditions of organs and tissues.

"But in pathology how vastly important, nay, how absolutely necessary, is an appeal to the microscope. How often are men, who have passed their lives in the examination of morbid structure, deceived in determining with precision the presence of inflammation or softening. Indeed, how can it be otherwise, when we consider the deceptive nature of the modes in which the investigation is determined? Thus, an intense degree of redness in a tissue is by some called congestion, by others inflammation. How vague are the ideas attached to the consistence of organs;—what appears healthy to one, seems to another somewhat indurated, and to a third softened. Again, it is impossible for such morbid anatomists to decide definitively, on the exact limits of any peculiar morbid structure. Who, for instance, can affirm, that in the brain, because the substance looks white and healthy, and neither softening nor induration be apparent, that it is in a normal state?

Not long ago, I saw a case which will illustrate this point. A man entered the Royal Infirmary, labouring under apoplexy. There was profound coma, stertorous breathing, full pulse, and all the signs of active hemorrhagic apoplexy. The whole right side of the body was completely paralyzed, in a state of resolution, the limbs, when raised, falling down, like inert masses. The whole of the left side, on the other hand, was intensely rigid, so much so, that it was impossible to flex either of the limbs. Dr. Spittal, who had charge of the case, diagnosed hemorrhage in the left, and inflammation in the right cerebral hemisphere. Notwithstanding the most judicious treatment the man died. On inspection, a large hemorrhagic effusion was found in the left hemisphere, so far confirming the diagnosis, and explaining the resolution and paralysis of the right side. In the right hemisphere, on the other hand, neither Dr. Spittal, nor myself, nor any of the assistants, could detect traces of inflammation. There existed, indeed, several small excavations, and the appearance which Dr. Sims has described as resulting from the cure of ramollissement; but nothing could be detected capable of explaining the severity of the symptoms. On examining it microscopically, however, I found the most evident traces of inflammatory action, and a new product formed in great abundance, to which Glüge of Brussels has given the name of globule of inflammation.

In several other cases, I have convinced myself by means of the miscroscope, that inflammation existed in textures where nothing abnormal could be seen with the naked eye, and in this manner have been enabled to explain many symptoms which otherwise would have remained inexplicable. A man died a short time since in the Infirmary, who had for several days been affected with loss of consciousness, and rigidity of the right arm. Besides a tumour in the brain, which had been previously diagnosed, I detected, by means of the microscope, inflammatory softening of the left corpus striatum, a lesion which, by unassisted vision, none present at the examination could positively assert to be present.

On the other hand, I have determined, in several cases, that lesions supposed to be inflammatory arose solely from hemorrhage, or simple congestion—that what has been considered tubercle was in fact infiltrated pus—that tumours imagined to be malignant, were really innocuous, and so on. Regarding all these points, we are enabled to get rid of the vagueness and looseness which at present prevail in connexion with them, and by means of the microscope, to arrive at positive information, on which the morbid anatomist may safely rely.

The microscope explains to us also why certain diseases are so intractable to treatment. It shews us, that several morbid lesions are composed of cells, each of which, as I have before stated, possesses an independent vitality. Thus, warts, melanosis, cancer, fungus hematodes, &c., defy the efforts of the practitioner, because he is unable to attack them through the organism of the individual in whom they exist. In fact, they are distinct beings, endowed with a vitality of their own, true parasites, which it is imposible to consider either as animal or vegetable, which feed upon the tissue they are found in, and can only, by its destruction or excision, be removed from the economy."

We must make one other extract to shew the applicability of the microscope to diagnosis.

" Mr. D———, an English gentleman, whose acquaintance I formed in Heidelberg, asked my advice for a complaint, consisting principally, as he stated, of headache, vertigo, and a disposition to faint, under which he had laboured for some weeks. He was thirty-five years of age, of a peculiarly sallow countenance; and though once robust and stout, was exceedingly weak, and greatly emaciated. He could not stand erect for any time, or stoop, without feeling faint. He had constant headach in the occipital region, sometimes extending to the forehead. He was very excitable; and the pulse, although generally slow and soft, would, on the slightest alarm, become rapid and thready. I learnt that he had been very dissipated, had suffered under several forms of syphilis, and had even then a stricture of the urethra, for which he had lately been treated by Professor Chelius. In addition to this affection of the urinary apparatus, he had for the last sixteen months laboured under what appeared to be an affection of the bladder. His urine was often cloudy, contained at different times more or less mucous flocculi, deposited a sediment which varied in amount, and exhaled a peculiarly fetid and disagreeable odour. He had consulted most of the celebrated physicians and surgeons in London. The former had told him that he laboured under disease of the kidney, and the latter, that he had chronic inflammation of the bladder. He even went to Leamington, to consult a celebrated physician of that town. He presented to me a mass of prescriptions, from which I learnt, that he had taken in turn mercury, arsenic, copper, opium, and all the heroic, as well as minor remedies. He had also been frequently leeched, and cupped in the loins; and even when I saw him, had an open issue in the lumbar region. He had always been kept on low diet, and was on his way to Carlsbad, to drink the purgative waters of that celebrated spring. I really did not know what to advise, and merely ordered him some emollient drinks, until I had further observed the case. One day I took home some of his urine, and examined it with the microscope. Much to my surprise, I found in it the spermatic animalcules in great abundance, some destroyed and broken down, but others entire, so that no doubt could exist regarding their presence. It was now apparent that the semen had found its way into the bladder, and the real nature of the case dawned upon me. On questioning him, I found that some time previously he had been conscious of involuntary seminal emissions. This had been increased by the antiphlogistic treatment pursued; and latterly there had appeared headache, fainting, and the other signs of irregular distribution of blood within the cranium. I immediately ordered the seton to be taken out of his back. Instead of his usual low diet, I ordered beef-steaks and good beer. At the same time, I administered vegetable and chalybeate tonics. Instead of keeping him confined to the room, I ordered him to take short walks, which were to be gradually increased in length, according to his improving strength. Instead of going to Carlsbad to drink purgative waters, I advised him to visit Wildbad, and use the stimulating and tonic carbonic acid baths of that spring. The cold douche to the back was also occasionally to be employed. The gentleman was intelligent, saw the force of my arguments, and followed the treatment proposed; and in six months I heard from him, that he was perfectly restored to health."

We may remark on the above case, that the "stimulating and tonic *carbonic acid baths of Wildbad*," exist only in the worthy doctor's own imagination, there being no carbonic acid baths there at all, and the waters themselves are as pure as the finest rock springs—the *temperature* (98°) being the quality on which their medical agency is founded. SCHWALBACH would have been a much more appropriate place to have sent the patient. But that is of no consequence. The case itself is curious—and we wish Dr. Bennett every success in his Histrological Lectures, which we recommend the students of Edinburgh to carefully attend.

---

ILLUSTRATIONS OF THE COMPARATIVE ANATOMY OF THE NERVOUS SYSTEM. By *Joseph Swan*. Part VII. (Last.) Price 7s. London, Longman and Co.

We cannot allow this work to be concluded without paying a just tribute to its author. He has devoted much time, great labour, and some pecuniary means to the practical elucidation of the nervous system. He has set himself to work to *dissect* it. The bent of one man's mind leans one way, that of another man's another, and we must take their contributions to the fund of human knowledge as we get them. We cannot quarrel with Adam Smith for not presenting us with an epic poem, nor with Milton for not writing a treatise on the wealth of nations. We accept the generalizations of Müller and the particular dissections of Swan, each good and useful in their way. And we only wish that the history of medicine contained fewer of the former and a less lack of the latter.

The part of the Illustrations before us contains plates of the facial nerve of the sow—the connexion of the cerebral nerves with the sympathetic of the same—the cerebral nerves of the jaguar—the sympathetic nerves of the sheep—the olfactory nerve of the horse—the sympathetic and other nerves in the head of the calf—the nervous system of the hypogastric plexus of the male-calf—the hypogastric plexus of the sow—the hypogastric plexus of the ass.

There follow some general and concluding observations, to some of which we shall direct attention.

*Cerebrum and Cerebellum in Animals.*—Mr. Swan tells us that the brain is more or less spherical or lobulated in all animals; in man, at the upper part of its hemispheres, there is an extensive fissure, and one more or less deep in many of mammalia; but in some of these and the other classes there is a very little, if any, separation. Convolutions answer a particular and not a general purpose; they are very deep in man, and some of mammalia: but in others and the several inferior classes they hardly exist. The great commissure is very extensive in man and such of mammalia as have the hemispheres high and large; it faintly exists in others, in which the lobes only just inclose the lateral ventricles; it is not present in the three lower classes. Ventricles vary in all the classes; the lateral has a posterior horn in simiæ proportioned to the posterior lobe, so that in some it is a mere chink; in birds it extends more posteriorly, at which part its parietes are very thin; it is placed anteriorly in amphibia and fishes. The third ventricle lies between the thalami in mammalia and birds: in birds it extends into the optic lobes; in amphibia and fishes it is continued from the same surface with the lateral. The fourth ventricle exists in all; and in birds, amphibia, and fishes, extends into the optic lobes and cerebellum. The transparent septum exists in mammalia only; in birds, the striated septum supplies the place of it and the great commissure. The former exists in mammalia only; in birds, the floor of the lateral ventricle supplies its place. The great hippocampus exists in mammalia but not in the other classes. The striated body exists in the three superior classes; in mammalia it is similar to that in man, but very different in birds and amphibia. The thalamus exists in the three superior classes, but is very small in amphibia. The soft commissure depends upon the presence of each thalamus; it is very tough in the turtle. The anterior commissure exists in the four, the posterior in the three superior classes. There is a pineal gland in mammalia and the turtle. The quadrigeminal bodies are distinct in mammalia, but vary, the nates being either larger or smaller than the testes; they are solid at birth; in birds they are flattened and large and have no distinction like that of the nates and testes, and contain a ventricle in each communicating with the third; they also exist as hollow bodies without any anterior or posterior separation in amphibia and fishes. The base of the brain is divided into lobes in man and simiæ, but in most others there is very little, if any, distinction; the pituitary gland exists in the four superior classes. Two distinct mamillary eminences exist in man, but they are very generally conjoined in mammalia; they do not exist in amphibia, they are however not only present but separate in fishes. The cerebellum exists in the four superior classes; in the invertebrated its presence is doubtful; it has large lateral lobes compared with the middle, and is large in proportion to the size of the body in man and simiæ; the lateral lobes compared with the middle ones are smaller in mammalia generally; it is convoluted throughout; in birds, it consists principally of a middle lobe, to which is attached on each side a small one like the lobule appended to the lateral lobe of the monkey and other animals; it is convoluted, and has a ventricle; in some of the amphibia, as the turtle, it is hollow, in several it is a mere rudiment. In the cod, it has a small ventricle, and consists principally of a middle lobe; in the skate it has a ventricle, it has also lateral lobes which are somewhat convoluted. The annular tubercle is largest in man, it is proportioned to the size of the crura of the cerebellum; it exists in mammalia, the trapezoid body is a resemblance of the posterior part of it; it is not distinct in birds, and does not exist in amphibia and fishes; it is proportioned to the size of the crura of the cerebellum. The oblong medulla is larger in mammalia in proportion to the size of the brain than in man, but particularly in the other classes; small olivary bodies exist in the monkey; the other eminences in the four superior classes are more or less indistinct.

*Spinal Marrow in Animals.*—The spinal marrow is nearly the same in the four superior classes: in the invertebrated there is a chord or ring in the place of it, analogcus to its nerves and ganglia; it varies either in breadth or length, according to the required motion of the spine, and the number and size of the nerves; it may be short and broad, or long and narrow, with enlargements in places from which larger nerves are to proceed: it may form a longer or shorter cauda equina. In birds it appears knotted, and has its dorsal part closely surrounded by bone, and has a lumbar ventricle; it reaches to the tail in birds, and generally in amphibia and fishes.

*Smelling* depends upon the olfactory nerve arising from the brain and the fifth arising from the oblong medulla. The organ may be small or very capacious; branches analogous to those of the fifth may produce a rudimental sense in some invertebrated animals.

*Seeing* depends upon the optic nerve arising from the brain, and the third, fourth, fifth, and sixth arising in the track of the oblong medulla. The organ varies in size, but not like the nose; the ciliary nerves are not in proportion to the size of the organ, but to the required powers of vision. Although there is an optic nerve arising from the brain in some of the invertebrated animals, yet in many instances the organ is rudimental and the nerve analogous to the ciliary.

*Hearing* depends upon the auditory nerve, the fifth, the hard portion and the glosso-pharyngeal, which arise from the oblong medulla. The auricle and tympanum vary in each class, and in different kinds of the same in a slighter degree. The labyrinth is similar in mammalia except slight variations in the windings of the cochlea; it is further modified in birds, and still more in amphibia and fishes. In fishes the auditory, or fifth, and glosso-pharyngeal are more conjoined for supplying the labyrinth; instead of the fifth and glosso-pharyngeal being confined to the tympanum and its appendages. In invertebrated animals the rudimental form of nerves may approach that of fishes and resemble that for the tympanum only in the higher classes.

*Tasting* depends upon the fifth, glosso-pharyngeal, and ninth, which arise from the oblong medulla. The organs concerned in its production are very extensive in many of mammalia. The nerves are proportioned to the organ and the oblong medulla, and not to the brain or cerebellum. Although branches of the fifth supply the mouth in the other classes, the glosso-pharyngeal appears to be the most important. In the invertebrated any sense of taste may have a rudimental condition approaching that in the three preceding classes.

*Sensation* depends upon the fifth and spinal nerves arising from the oblong and spinal medulla; the ganglia attached to them are variously constructed in different animals. The brain may be large or very small; the cerebellum may be large or rudimental. In invertebrated animals the nerves may proceed from the subœsophageal ganglion and the prolongation of this in a chord or ring, or from ganglia having the least possible resemblance to a brain. It is probably modified by all these changes as well as by the extent of the convolutions of the brain. Under this head the following parts may be included: skin, teeth, bone, ligament, and those giving origin to horn, hair, and nails.

*Voluntary Motion* is under the third, fourth, fifth, sixth, hard portion, ninth, accessary, and spinal nerves, and the oblong and spinal medulla. In invertebrated animals the nerves may proceed from the subœsophageal ganglion, or from a prolongation of this in a chord or ring, or from ganglia having the least possible appearance of a brain; in many instances there is no difference in the dorsal and ventral surfaces of the chord or ring, and in the highest of this class there is not the same distinction as in the vertebrated.

*Involuntary Motion* is under the sympathetic, or such of the common motive as have their powers directed by the action of moving parts placed in contact or connexion with them; it is therefore principally under the influence of the oblong and spinal medulla.

*Circulation.*—The nerves promoting it proceed from the sympathetic and par vagum, so that it is principally under the influence of the oblong and spinal medulla. In mammalia and birds which have hot blood and a completely double circulation there is a large brain. In animals with cold blood, as amphibia which have not a completely double circulation, and fishes which have one still more simple, the brain is small and the cerebellum may be a mere rudiment. In invertebrated animals there may be a different form of the circulatory organs when the nervous system is similar.

*Spinal Marrow.*—It gives origin to the sensitive and motive nerves of the trunk of the body; its functions are very limited, independently of the brain. In invertebrated animals, a long chord or ring analogous to the ganglia and nerves of the spinal marrow and sympathetic exists.

*Oblong Medulla.*—It is required for all the vital functions, and for sensation and motion; the brain, cerebellum, and spinal marrow are not absolutely necessary, they nevertheless, according to their development, extend its powers. Only the two organs of smelling and seeing receive nerves directly from the brain, and they require others from the oblong medulla for the completion of their functions. All the other organs, not supplied by nerves from the oblong and spinal medulla, are indirectly connected with those parts through the sympathetic nerve. If the brain and cerebellum do not immediately promote the functions of the several organs, they complete the concatenation of faculties required in the more perfect creatures.

*Brain.*—Only a very small brain in proportion to the size of the body exists in some animals, and is principally for the senses of smelling and seeing, and for ministering to the intellect, and for this purpose it is fashioned with modifications of structure. According to the increase of the intellectual faculties, the hemispheres become larger in proportion to the oblong medulla. In invertebrated animals, its most complex state is inferior to that of fishes, and in many instances is so rudimental as scarcely to deserve the name.

*Cerebellum.*—Its condition may be a mere rudiment in some complicated animals, and increasing from this to its large size in man. The vital and instinctive functions, and those of sensation and voluntary motion, do not depend upon it.

*Sympathetic Nerve.*—It exists more or less distinctly, but with modifications, in the four superior classes; in the invertebrated, the functions usually performed by it are more conjoined with the rest of the nervous system. It is related to the spinal and oblong medulla, through the nerves arising from these parts.

Mr. Swan alludes to the soul, and to the instinctive essence. We confess that he does not succeed in clearing up the mystery surrounding them.

*Grey and White Matter.*—Neither the grey nor white matter can be discovered in every animal; but as they exist so extensively, they may be considered as one of the chief means of combination with the nervous element, and thus as a principle.

*Shapes of the Grey and White Matter.*—The grey and white matter require to be placed in a particular order, shape or form, and to be more or less intermixed with each other; and according to this elaboration are their powers varied and modified, either for general or particular centres. The more extended portions of the brain for promoting the intellectual faculties, and the more particular ones as centres for the origins of the nerves, are formed on an appropriate plan, and have their prescribed situation, but vary in some respects in different animals. The instinctive essence manifests its power differently, according to the condition of the structures though which it acts. The more elaborate the ranges of the fibres of the centres, and the construction of the several organs of the body through which the mind and instinctive essence are approached, the more exalted is the manner in which perceptions and the commands of the will are executed; so that, on account of the great development of the brain in man, the mind receives and imparts impressions, which the instinctive essence cannot. The shapes may, therefore, be reckoned as a principle, as the modifications of the same determine the extent of many faculties

We cannot take leave of the work without again commending the unwearied diligence of Mr. Swan, nor without holding up for our readers' emulation both the book and its author.

---

THE DOUBLE-FLAP AND CIRCULAR AMPUTATIONS CONTRASTED; BEING AN ABSTRACT OF A FIRST PRIZE ESSAY AT THE UNIVERSITY OF EDINBURGH. By *F. N. Machardy*, A.M. and M.D., Surgeon. London, Simpkin, and Co., 1841.

WE do not know that we ever saw exactly such an Essay. It is as full of quotations as Burton's Anatomy of Melancholy—of errors of the press as a "last dying speech and confession"—and of novel turns in grammar as a nursery-maid's love letter. Withal, there is much information in it, and a vast deal of labour has been spent on it.

Dr. Machardy leans, and most Scotch surgeons do, to the flap in preference to the circular operation. He urges arguments of all sorts in its favour. These we shall pass over, for the purpose of introducing his statistical facts, which are highly deserving of attention.

### AMPUTATION OF THE ARM.

Dr. Machardy presents the results of thirty recorded circular amputations, and twenty-four flap ones.

1. *Circular Operation.*—Mr. Hargrave observes, that amputation of the humerus by the circular incision is confined to the space between the elbow and the insertion of the pectoralis major; such selection being apparent from the nature of this operation requiring an equalized distribution of muscular tissue surrounding the limb.

Key and Cooper, at Guy's; Guthrie and White, Westminster; Travers, St. Thomas's; Hawkins and Keate, St. George's: Latta and Hunter, Edinburgh:

and Cooper, at Glasgow Infirmaries, have severally amputated the arm by the circular incision, the results of which are now subjoined:

Of xxx amputations,

1 had Un. by Suppurat.; 2, second Hem.; 3, Phlebit.; 1, Erysipelas; 6, Gang.; 1, Abscess; 4, Spasm; 3, Conical Stumps; and 5, Necrosis; 4, Resected Circular Stumps.

Generally, 4 ligatures were applied.

Time of cure, 14 to 60 days.

Majority, chronic cases.

Average age, 20.

Deaths, 9. 3 from immediate amputation, 2 of which were succeeded by phlebitis in debilitated cases, æt. 11 and 18; 1 phlebitis and another erysipelas, succeeding amputation for gangrene, æt. 18 and 72; 1 scrofulous disease, æt. 11; 1, gangrene, æt 58; 1, diseased lungs; and 1 from the effects of the operation, æt. 63.

2. *Flap Operation.*—Syme and Lizars conceive, that if this method is applicable anywhere on the body, it is between the elbow and shoulder joint, and within these limits has the former and Dr. Busche found it successful, in cases of emaciated individuals, where there was flaccidity of musclar fibre.

Key, Morgan, and Bransby Cooper, at Guy's; Liston, and Mr. Cooper, at the North London; Wardrop, at the Hospital of Surgery, Panton Square; Ballingall, and Syme, at Edinburgh: and Messrs. Weir, and Perry, at Glasgow Infirmaries, have amputated by the double flap, as well as Klein, Gräefe, and Langenbeck, who, by such a system, have made admirable stumps, with results as follow:—

Of xxiv amputations,

5 Un. by Suppurat.; 2, Sec. Hem.; 1 Phlebit.; 2, Erysipelas; and 4 Gangrene.

Generally, 4 ligatures were applied.

Time of cure, from 18 to 22 days.

Majority, chronic cases.

Average age, 30.

Deaths 5. 2 from secondary amputation, the one being followed by phlebitis, the other gangrene, æt. 55: 1 from the effects of gangrene causing amputation, æt. 20; and two from gangrene attacking the stump.

Klein, Liston, and Cooper, have advocated antero-posterior flaps, which, though of general application, still possess the disadvantage of favouring a protrusion of bone, when formed at the insertion of the deltoid muscle; but by there adopting the lateral flaps, as employed by Garengeot, and Mr. Morgan at Guy's, this occurrence will be entirely prevented.

Hey deemed the flap unnecessary in the arm, and many operators are of opinion that a sufficient stump can be effected by the circle; but even here there are living monuments of *its* deficiency, such as have never yet been recorded of the flap.

### Amputation of the Fore-Arm.

At Guy's, Key, and Bransby Cooper; St. Bartholomew's, Lawrence, and Sir Anthony Carlisle; and at the Edinburgh Infirmary, Latta, have amputated the fore-arm by the circular operation, the result of whose experience with that of others is now detailed:—

Of xxvi amputations,

2 had Un. by Suppurat.; 1, Sec. Hem.; 1, Phlebit.; 2, Spasms; and 1, Conical Stump.

Generally, 3 to 5 ligatures were applied.
Time of cure, 9 to 20 days.
Majority, chronic cases.
Average age, 20.
Deaths, 2. 1 from phlebit. following amputation for gangrene, æt. 21.; the other from hæmorrhagic disease, æt. 39.

By Key, and Bransby Cooper, at Guy's: Lawrence, St. Bartholomew's; Wardrop, Hospital of Surgery, Panton Square; Latta, Ballingall, and Syme, Edinburgh; and Messrs. Cooper and Perry, at Glasgow Infirmaries, has the flap been employed with the following results:—

Of xxvii amputations,
2 had Un. by Sup.; 1, Sec. Hem.; 2, Gang.; and 1 Cystitis.
Generally, 2 or 3 ligatures were applied.
Time of cure, 7 to 25 days.
Majority, chronic cases.
Average age, 25.
Deaths, none.

### Amputation of the Thigh.

1. *Circular Amputation.*—At La Charité, Kuhk; Guy's, Sir Astley Cooper, Key, Keate, and Morgan; St. Bartholomew's, Lawrence, Vincent, Lloyd, Stanley, Earle, and Keate; Middlesex, Bell and Mayo; Westminster, Guthrie and White; St. George's, Brodie, Keate, Jeffreys and Hawkins; St. Thomas's, Sir C. Blike and Green: and, at the London Hospital, Sir William Blizard and Mr. Andrews, have employed the circular incision on the thigh; with what success the following statistics exhibit:—

Of c amputations,
20 had Un. by Sup.; 9, Sec. Hem.; 3, Phlebit.; 2, Erysip.; 7 Gang.; 4, Cystitis; 7, Spasms; 5, Con. Stump; and 10 Necrosis.
6 Resections of circular stumps.
Generally, 2, 5, 6, 8, 9, 12, and 16 ligatures were applied.
Time of cure, 14, 20, 25, and 60 days.
Majority, chronic cases.
Average age, 30.
Deaths, 28. 6 from secondary amput., 1 followed by gang., respective æts. 2, 26, 44, 45, 57, and 64; 1, immed. amp. drayman, æt. 42; 1, effects of operation for gang. æt. 52; 1, gang. lax fibre, æt. 52; 2, suppuration, 1 in arteries, the other in stump, æts. 4 and 12; 1, sec. hem.; 6, irritable stump, inducing secondary disease, æts. 20, and upwards; 3, phlebit. æts. 30, upwards; 2, hectic fever; 1, gout; 1, general injury previous to operation; 2, scrofulous diathesis; and 1 from the effects of resection.

2. *Flap Amputation.*—Though Dupuytren declared, that the flap operation in the trunk of a member had for long been banished from modern surgery, yet its revival in the thigh seems well established by Roux at La Charité; Key, Morgan, and Bransby Cooper, Guy's; Vincent, St. Bartholomew's; Mayo, Middlesex; Sir Anthony Carlisle, Westminster; Brodie, St. George's; Walker and Green, St. Thomas's; Luke, London; Liston, North London; Ballingall, Syme, and Lizars, Edinburgh; Drs. Buchanan, and Macfarlane, Messrs. Davidson, and Perry, Glasgow Infirmaries, with the subscribed results:—

Of cii amputations,
13 Un. by Sup.; 9, Sec. Hem.; 8, Phlebit.; 1, Ery.; 9, Gang.; 4, Cyst.; 3, Partial Con. Stumps, oc. by disad. in cure; and 2 Nec.; 4, Flap Resections of Circular Stumps; and of 1 Flap Stump, occasioned by phagedenic ulceration.

Generally, 2, 3, 4, 5, and 15 ligatures were applied.

Time of cure, 10 to 27 days.

Majority, chronic cases.

Average age, 25.

Deaths, 28. 5 from second. amput., gang. supervening on 2, and phlebit. on one, æts, 50, 56, 58; 1, immed. amput. and general bruises; 3, loss of blood depending on operation, one in which no arteries were tied, æts. 10, 20; 8, phlebit. æts. 11 to 32; 2, gang. appearing previous to the operation in one, and subsequent in the other, æts. 18 and 40; 1, effects of necrosis previous to amputat., æt. 10; 1, excessive suppurat.; 1, erysip.; 2, operation for compound fracture, æts. 40 and 58; 1, abscess; 1, exhaustion, æt. 31; and 2 from fever, æts. 34 and 36.

### Amputation of the Leg.

1. *Circular Amputation.*—By M. Cloquet, at Hôpital de l'Eisle; Morgan, Key, and Bransby Cooper, Guy's; Sir Charles Blike, Lawrence, and Earle, St. Bartholomew's; Guthrie, Westminster; Travers and Hawkins, St. George's: and Messrs. Macfarlane and Weir, Glasgow Infirmaries, the circular division of the leg has been performed; and although Bromfield, Syme, and Mr. Langstaff have instanced woeful results from this operation, yet Latta and Sir Astley Cooper have found it very successful. However, the following numerical results may aid the illustration:—

Of xliii Amputations,

9 had Un. by Sup.; 3, Sec. Hem; 2, Phlebit.; 2, Erysip.; 7, Irrit. or Spasms; 6, Con. Stump; 6, Gang.; and 1, Necrosis.

3, C. Rections.

Generally, 3 to 6 ligatures were applied.

Time of cure, 21 to 50 days.

Majority, chronic cases.

Average age, 35.

Deaths, 7. 2 from Amput. for Comp. fract. tending to Gang. æts. 60 and 21; 1, Amput. for Gang. æt. 52; 1, immed. Amput. Gang. supervening, æt. 17; 2, Amput. for dislocations, Gang. attacking the stumps, æts. 60 and 53; 1, excessive Suppurat.; and 1, Second. Amputat.*

2. *Flap Amputation of the Leg.*—The lateral double-flap operation, as practised by Key at Guy's; White, Westminster; Travers and Green, St. Thomas's; Wardrop, Hospital of Surgery, Panton Square; Syme, at Edinburgh; and Macfarlane, at the Glasgow Infirmaries, has now almost been laid aside even by Roux, who revived it, and has given place to the antero-posterior flaps, or, more

---

* These results, meagre as they may be, confirm the views in favour of primary amputation, so ably and justly advocated by Thomson (Obs. on Hosp. in Belg. 1816,) Cooper, Lawrence, Guthrie, Buchanan, Dupuytren, Boucher (Mém. de l'Acad. de chir. t. 2, 1753, p. 461,) and Larrey (Mem. de chir. milit. t. 1 et 3, p. 349.) By primary amputation, Percy, at the head of the military surgery in France, lost 6 out of 92, or about 1 in 15; Lucus, 5 out of 75, or 1 in 15; and, on the last days of July, all were cured, generally in the space of 25 days. Of secondary, 7 died out of 11 at Glasgow Infirmary (Glas. Med. Jour. v. 3 and 4, pp. 113, 220): Pelletan, at the head of the Civil Hospitals in Paris, lost 5 out of 6, or 1 in every 1 and one-fifth; and similar results have been observed by C. T. Kuhk at Hôpital La Charité; and at Meath Hospital, Dublin, as is learned from its quarterly reports.

strictly, the *single flap* of Dupuytren or Liston, to the former of which, however, viz. the lateral amputation, the following results apply:—

Of xii Amputations,

1 had Un. by Sup.; 1, Sec. Hem.; 3, Phlebitis; and 2 Sinus.; 1 Resect. of Cir. Stump.

Generally, 1 to 3 ligatures were applied.

Time of cure, a few days.

Average age, 30.

Deaths, 4. 3 from Phlebit; and 1 from Gang. æts. 35, 47.

### GENERAL COMPARISON OF THE TWO OPERATIONS.

| Out of 199 cases by the circular incision, | | | Out of 165 cases by the double flap, | |
|---|---|---|---|---|
| 42 | or 1 in every 4.73- | had Un. by Suppurat. | 21 | or 1 in every 7.85- |
| 15 | 13.26- | Sec. Hem. | 13 | 12.68- |
| 9 | 22.11- | Phlebitis | 12 | 13.75 |
| 5 | 39.8 | Erysipelas | 3 | 55 |
| 19 | 10.47- | Gangrene | 15 | 11 |
| 5 | 39.8 | Abscess | 7 | 23.57- |
| 19 | 10.47- | Spasm | None. | |
| 10 | 19.9 | Conical Stump | 3 | 55 |
| 16 | 12.43- | Necrosis | 2 | 82.5 |
| 16 | 12.43- | Resections | 1 | 165 |
| 156 | 1.27- | Absolute ratio | 77 | 2.14- |
| 46 | 4.32- | Total deaths | 37 | 4.45- |

This then, says our author, is experience, without which, as Lock and Bacon have declared, no science can be complete; and if these facts are as true as they have been impartially collected, they evidently show the *double* superiority of the flap over the circular incision.

These are the statistical data collected for us. They reflect, if accurate, which we presume they are, very great credit on the industry of their compiler. But we do think him rather hard on the "Circulists" and Circular Amputation. He badgers them unmercifully. In fact they can't survive it, and we soon shall hear of circular amputations as obsolete samples of the wisdom of our ancestors.

"Just as if better stumps could be formed by the circular incision, which, though evidently entitled to the appellation of 'plump,' still, generally bear no small resemblance to a ship amputation, where the Captain operated as another read; so by the alternate use of theory and practice, the limb was dismembered in the course of a day, leaving for years, in every sense, rotundity of parts, but an almost denuded bone."

"Analogous to those cases of extensive burns on the neck, where the cure was deemed complete, yet the chin left bridled to the breast, are the instances of circular amputations of the thigh, condemned to the Cripplegate Workhouse, for twenty years under the superintendence of Mr. Langstaff, in whose museum relics yet remain, emblems of unphilosophic surgery—of unphilosophic men!"

Our author winds up:—

"Just as three memorable eras have arisen in surgery—the invention of the ligature by Ambrose Paré—the tourniquet by Louis Petit—and the flap by Loudham and Yonge—so have torsion and styptics made attempts to subvert the first, manual compression the second, and an incorrigible ancestral adherence to the circular incision the last. Yet each have been fraught with blessings to mankind—each has revived the Syracusan ejaculation—Εὕρηκα! Εὕρηκα!"

